# Comparison of Clinical Efficacy between Arthroscopic and Open Surgery for Ogden Type 1–2 Peroneal Tendon Dislocation

**DOI:** 10.1111/os.14035

**Published:** 2024-03-21

**Authors:** Zhenyu Wang, Guo Zheng, Fangcheng Yang, Yuanqiang Li, Yang Liu, Xinyu Xie, Xu Tao

**Affiliations:** ^1^ Sports Medicine Center The First Affiliated Hospital of Army Medical University Chongqing China

**Keywords:** Arthroscopic Surgery, Open Surgery, Peroneal Tendon Dislocation, Superior Peroneal Retinaculum

## Abstract

**Objective:**

While the incidence of peroneal tendon dislocation (PTD) is relatively low, it is frequently underdiagnosed in clinical practice, and the misdiagnosis or improper treatment of this condition may lead to a decline in patients' quality of life. Currently, the surgical treatment options for PTD mainly include open and arthroscopic surgery. However, in order to evaluate the advantages and disadvantages of these two surgical approaches, further comparative research is needed. Therefore, the aim of this study is to investigate the early clinical outcomes of arthroscopic and open surgery in the treatment of Ogden type 1–2 PTD.

**Methods:**

We conducted a comprehensive analysis of 46 patients diagnosed with PTD who underwent surgery at our institution between January 2017 and January 2023. The patients were divided into two groups: the open surgery group, consisting of 26 cases, and the arthroscopic surgery group, consisting of 20 cases. To compare the effectiveness of the surgical approach, we evaluated several parameters, including the integrity of the superior peroneal retinaculum on MRI images, functional scores, pain interference scores, and ankle eversion muscle strength. These assessments are conducted respectively before the surgery, 1 month after the surgery, 3 months after the surgery, and at the final follow‐up for each group of patients (at least 6 months post‐surgery). Demographics and intergroup comparisons of the two groups of data were analyzed by t‐test or the Mann–Whitney U test. Intragroup comparisons of the two groups of data were analyzed by one‐way analysis of variance (ANOVA) or the Kruskal–Wallis test, followed by post hoc multiple comparisons.

**Results:**

In the intragroup comparisons, both the arthroscopic surgery and the open surgery group demonstrated significant improvement in functional scores, pain interference scores, muscle strength, and MRI findings at the final follow‐up postoperatively (p < 0.01). However, the open surgery group exhibited significant improvements in these outcomes at the final follow‐up, while the arthroscopic surgery group showed significant improvement at 3 months postoperatively. In intergroup comparisons, the arthroscopic surgery group outperformed the open surgery group in functional scores, pain interference scores, and muscle strength 3 months after the surgery, with statistically significant differences (p < 0.01).

**Conclusion:**

Arthroscopic surgery offers advantages in early clinical outcomes, such as pain relief, function, and muscle strength improvement. However, over time, both approaches provide similar results regarding effectiveness.

## Graphical abstract

This study compares the early clinical outcomes of open and arthroscopic surgery to treat peroneal tendon dislocation. The study included 46 patients divided into two groups. Both groups showed improvement in functional scores, pain interference scores, muscle strength, and MRI results. The arthroscopic surgery group showed superior results in functional scores, pain interference scores, and muscle strength 3 months after surgery. However, both surgical approaches have similar long‐term effectiveness.

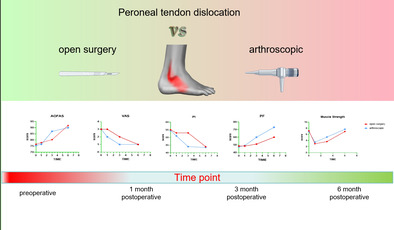

## Introduction

Peroneal tendon dislocation (PTD) is a relatively rare sports injury, accounting for 0.03%–0.5% of all ankle injuries. It occurs when the peroneal tendon breaks through the superior peroneal retinaculum (SPR) and escapes from the fibular groove, leading to external ankle instability, pain, and other clinical symptoms.^1^ It is commonly seen in high‐impact activities such as alpine skiing, rugby, tennis, basketball, football, skating, and mountaineering. It is commonly associated with fractures or dislocations of the fibula, calcaneus and talus, as well as lateral ankle sprain.^2^ The peroneal tendon is an important component for maintaining ankle joint stability. Following dislocation, the peroneal tendon fails to adequately support the stability of the ankle joint, resulting in lateral ankle instability, tendon rupture, degeneration, tendon pathology, and the development of complications such as chronic pain.^3^ These issues can negatively impact walking, running, and other daily activities. Additionally, misdiagnosis of acute PTD can lead to chronic cases as surgical specialists may focus more on treating fractures or sprains and overlook tendon‐related problems. Therefore, early diagnosis and appropriate treatment are crucial in order to mitigate the consequences.^4^

In clinical practice, the Ogden classification is commonly used for PTD. The classification includes: Type I—The SPR is partially elevated off of the fibula (fibrocartilaginous ridge remains intact) allowing for subluxation of both tendons; Type II—The SPR is separated from the cartilofibrous ridge of the lateral malleolus, allowing the tendons to subluxate between the SPR and the fibrocartilaginous ridge; Type III—There is a cortical avulsion of the SPR off of the fibula, allowing the subluxated tendons to move underneath the cortical fragment; Type IV—The SPR is torn from the calcaneus, not the fibula.^5^ Types I and II are the most commonly encountered in clinical practice and are also associated with milder injuries. Previous studies have shown that arthroscopic surgery has achieved good clinical outcomes in the treatment of Ogden Type I and II PTD, facilitating early return to physical activity for patients.^4^, ^6^ However, the advancements in arthroscopic techniques have sparked debates among clinicians regarding the comparative efficacy of arthroscopic surgery versus open surgery, with specific considerations as follows: Efficacy Comparison: While particular research indicates that outcomes of arthroscopic surgery may be on par with those of open procedures, dissenting studies suggest that open surgery may more effectively restore the stability and functionality of the peroneal tendons. Operational Risks: Arthroscopic surgery is characterized by its minimally invasive nature, requiring smaller incisions and usually leading to shorter durations of postoperative recovery.

Despite these advantages, similar to open surgery, there is also an inherent risk of complications such as nerve or vascular injury. Further comparative studies are still needed to validate the advantages and disadvantages of the two surgical procedures.^4^, ^7^ The current study retrospectively analyzed the clinical data of 46 patients who underwent either arthroscopic or open surgery for Ogden 1–2 type peroneal tendon dislocation. Our research objectives encompass three main aspects: (i) Independently validating the clinical efficacy of open surgery and arthroscopic surgery as distinct surgical approaches; (ii) Comparing the differences between the two surgical methods within the same timeframe; (iii) Providing a detailed exploration of the characteristics of both surgical techniques.

## Methods

### Patients

This study was approved by the ethical review of our hospital ((B)KY2023151), and informed consent was obtained from all patients. Patients diagnosed with Ogden 1–2 type PTD in our hospital from January 2017 to January 2023 who underwent either open or arthroscopic surgery performed by the same team of doctors.

**FIGURE 1:**
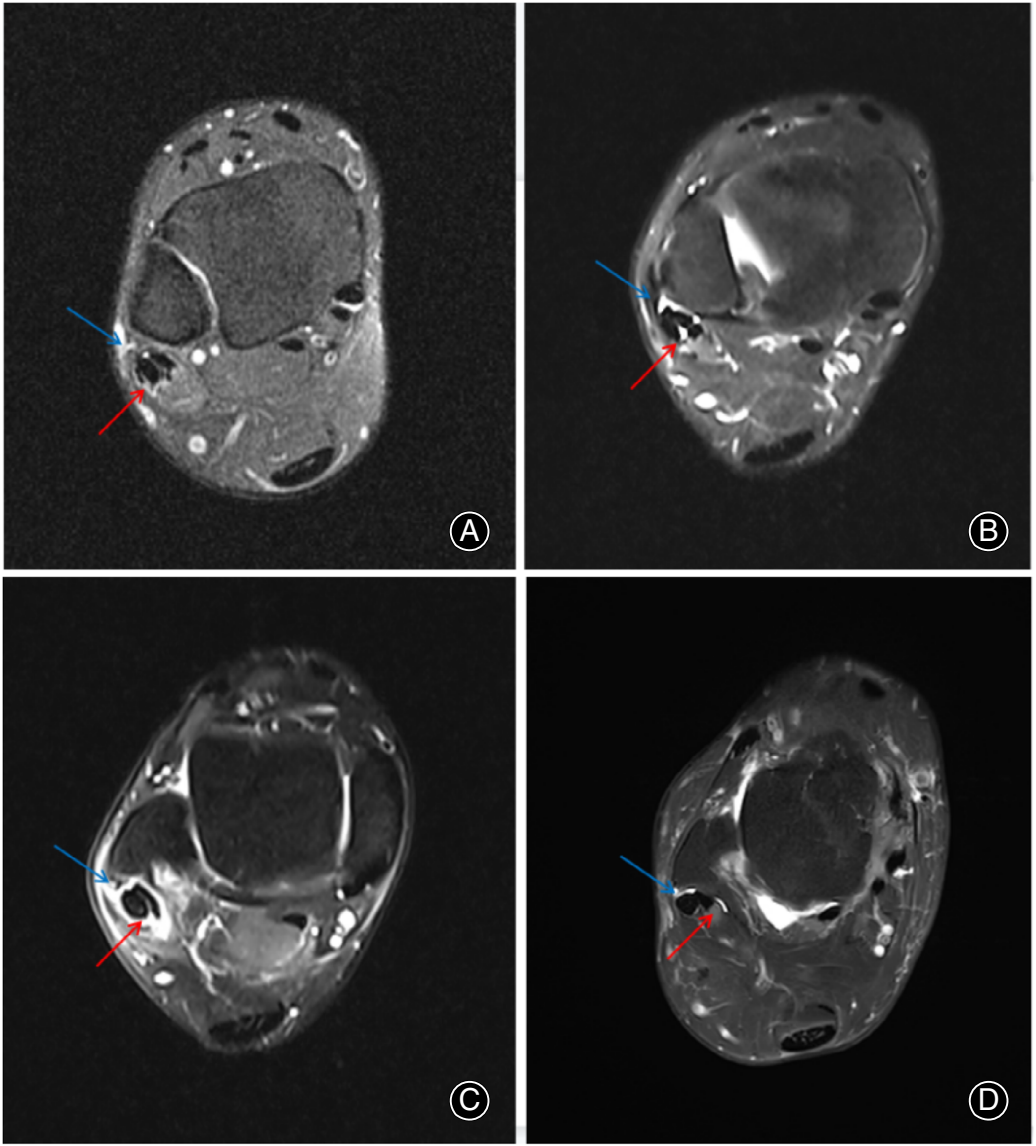
(A) SPR rupture (blue arrow), peroneal tendon injury (red arrow); (B) SPR rupture (blue arrow), peroneus quartus muscle (red arrow); (C) SPR rupture (blue arrow), peroneal tendon sheath edema (red arrow); (D) SPR rupture (blue arrow), low‐lying muscle belly of the PBT (red arrow).

**FIGURE 2:**
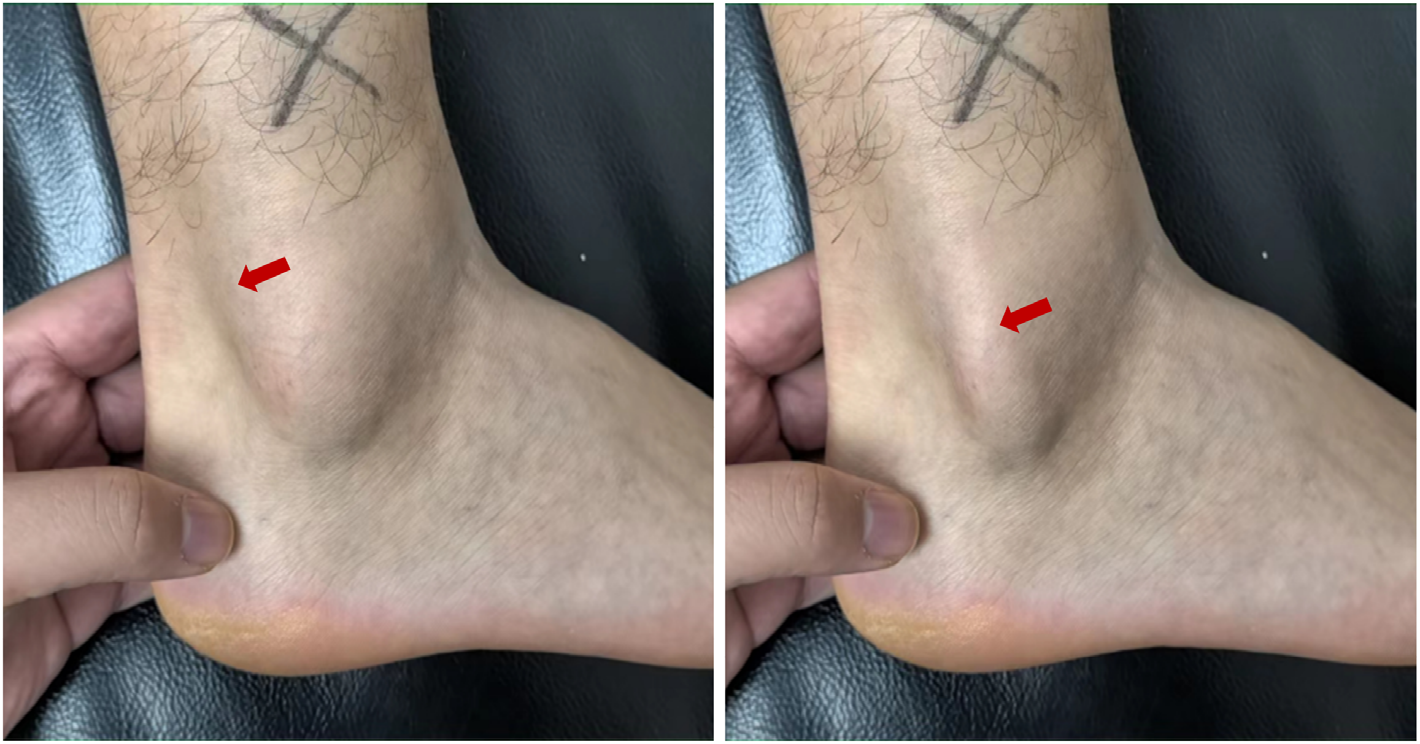
Physical examination of the PTD (the red arrow is the peroneal tendon).

Inclusion criteria: (1) Patients diagnosed with Ogden type 1–2 PTD; (2) Underwent either arthroscopic or open surgical treatment, which included SPR repair, resection of the low‐lying muscle belly of peroneus brevis tendon (PBT), and groove deepening; (3) Received conservative treatment for at least 3 months prior to surgery; (4) The follow‐up time exceeds 6 months. Exclusion criteria: (1) Patients who were treated for other foot and ankle ligament injuries in addition to PTD; (2) Patients with incorrect hindfoot alignment; (3) Patients with gout or rheumatoid arthritis. (4) The patient has a history of previous foot or ankle fractures; (5) There are neuro‐muscular factors contributing to weakness of the peroneal tendon. We compared the demographic and baseline clinical data of patients undergoing the two surgical procedures, including age, body mass index (BMI), time of illness, time of follow‐up, and surgical time. In this study, preoperative physical examination and MRI were used to assess the injury to the SPR and PTD and determine if there were any lesions in the peroneal tendon (Figures 1 and 2).

### Surgical Method

Anesthesia and position: patients of two groups underwent surgery in a lateral position under either nerve block anesthesia or general anesthesia. A thigh tourniquet was inflated to 300 mmHg to provide a bloodless surgical field.

#### Open Surgery Procedure

**FIGURE 3:**
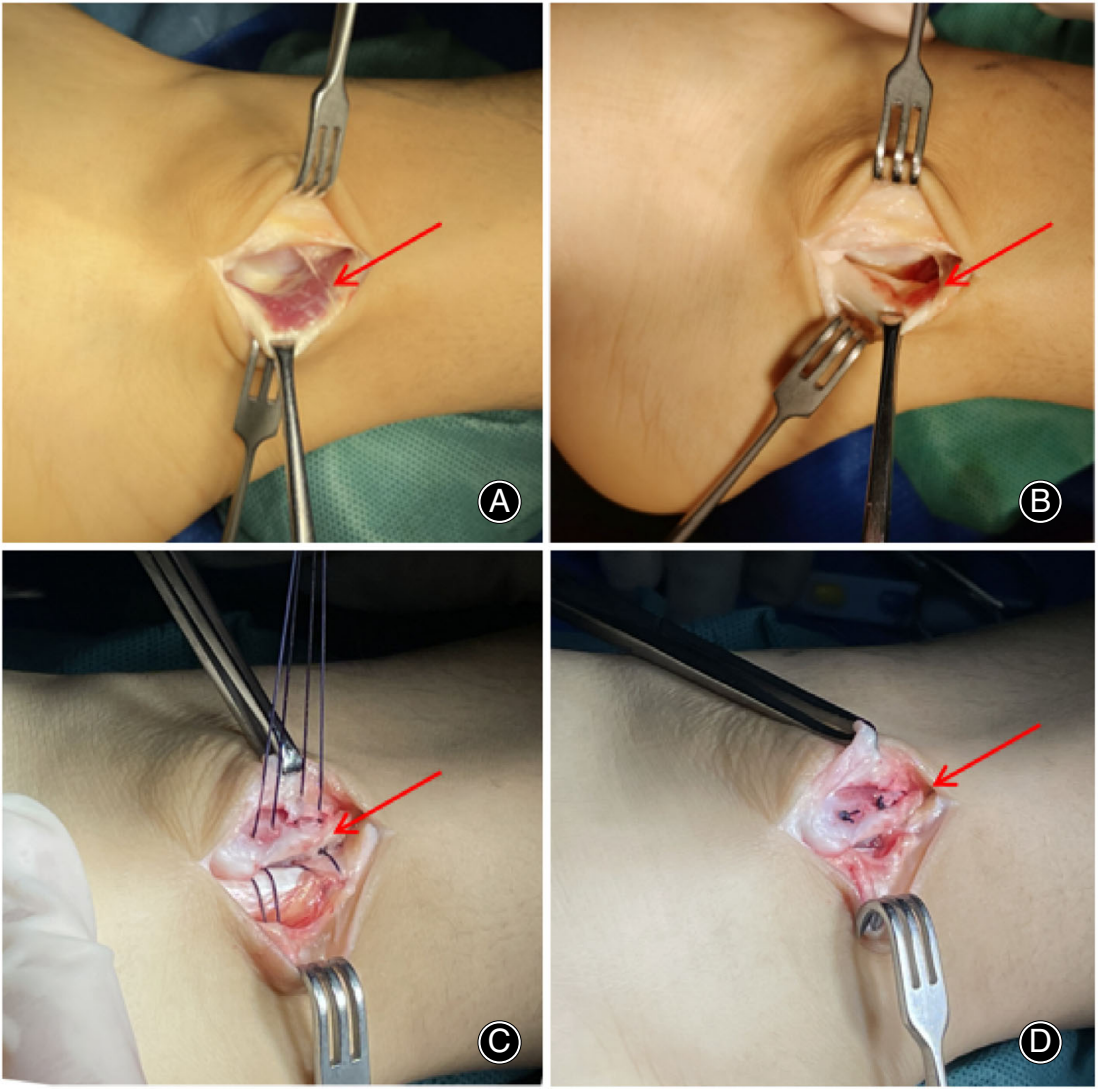
Open surgical procedure: (A) Low‐lying muscle belly of the peroneus brevis tendon; (B) Resection of the low‐lying muscle belly; (C) A 1.5‐mm tunnel was made on the fibular side, and the SPR was sutured behind the fibular ridge; (D) The stitches were tightened and knotted.

1. Approach and Exposure: The open surgery procedure commences with a 5 cm surgical incision made at the posterior inferior aspect of the fibular bone. 2. Excision of peroneus quartus muscle and pseudocyst: The surgical incision allows for the opening of the SPR enabling the resection of the inflamed tendon sheath, the peroneus quartus muscle and the low‐lying muscle belly of the PBT. The excision ranging from 2 cm above the SPR to the fibular groove. Additionally, the pseudocyst is also excised. 3. Fixation of SPR: A surgical thread is passed through the outer posterior edge of the fibula, and the SPR is secured to the posteromedial side of the fibular ridge. 4. “Vest over pant” technique and groove depth: To strengthen the periosteum and prevent the formation of pseudocysts, the “Vest over pant” technique is employed.^8^ If the fibular groove is shallow, grinding is performed to optimize its depth. Finally, the skin is sutured, and plaster is applied for fixation (Figure 3).^9^, ^10^

#### Arthroscopic Surgery Procedure

**FIGURE 4:**
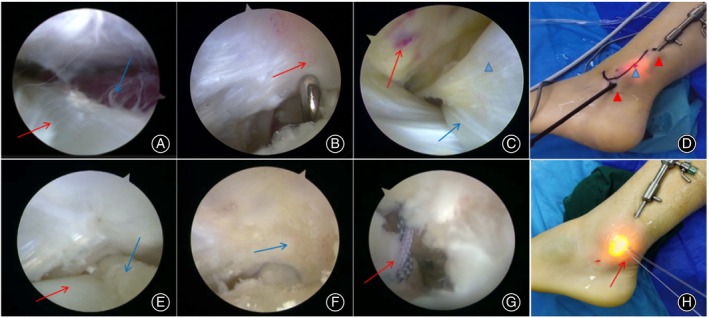
Arthroscopic surgical procedure: (A) Peroneus longus tendon (red arrow) and low‐lying muscle belly of the peroneus brevis tendon (blue arrow); (B) Flat fibular groove (red arrow); (C) SPR injury (red arrow), peroneus longus tendon (blue arrow), and peroneus brevis tendon (blue arrowhead); (D) Arthroscopic operation and observation portal (red arrowhead), suture anchor placement portal (blue arrowhead); (E) Peroneus brevis tendon (blue arrow) and peroneus longus tendon (red arrow) after resection of the low‐lying muscle; (F) After fibular groove formation (blue arrow); (G) SPR onto suture anchor (red arrow); (H) Anchor suture (red arrow).

1. Portals Placement: Two portals are placed along the peroneal tendon sheath. The distal portal is positioned just below the tip of the lateral malleolus, while the proximal portal is located ~4 cm above the distal portal and near the SPR. The precise positioning of the proximal portal is determined using arthroscopic guidance. 2. Tendon Examination: The peroneal tendons are carefully examined for signs of inflammation (tenosynovitis) or tears. Arthroscopic synovectomy, debridement, or repair of damaged tendons is performed as necessary. 3. Muscle Belly Resection: Under arthroscopy, the low‐lying muscle belly of the PBT is resected using a shaver. This step involves removing any muscle tissue that may impede the smooth movement of the tendon. 4. Groove Assessment and Grinding: The fibular groove is assessed to evaluate its shape. If the groove exhibits a typical arc, no further intervention is required. However, if the groove appears flat or protruding, grinding creates a smoother groove, allowing the tendon to move more freely.^11^ 5. SPR examination and repair: The integrity of the SPR was assessed during the arthroscopic surgery procedure. Along with the assessment, any elevation of the SPR was observed and noted. A suture anchor portal is created at the site of SPR injury under arthroscopy. A 2.9‐mm Mitek suture anchor (Depuy, Johnson & Johnson Company) was inserted into the anterior fibular ridge and sutured with the SPR to prevent future PTD during ankle joint movement. 6. Closure: After completion of the procedure, the surgical incisions are sutured, and the ankle is immobilized using a plaster cast (Figure 4).^4^, ^12^

### Rehabilitation

After PTD surgery, plaster immobilization is required for the first 4 weeks. During this immobilization period, cold compresses and limb elevation are the primary methods for mitigating pain and swelling. Concurrently, straight leg raises should be performed to prevent muscle atrophy in the lower limbs. In postoperative weeks 5–6, the plaster cast is replaced with an adjustable ankle‐foot orthosis. Weight‐bearing exercises are initiated, mainly focusing on muscle stretching and range of motion exercises. By weeks 7–8, patients can progress to full weight‐bearing, jogging, and complete balance and coordination exercises such as single‐leg stands. From postoperative weeks 9–12, intensive training continues, increasing the intensity and complexity of rehabilitation exercises. This phase mainly aims to restore the ability to perform jumping and explosive activities and gradually participate in low‐competitive sports. The rehabilitation protocol post‐PTD surgery is tailored based on the surgical approach and the integrity of the SPR. Generally, for open surgical procedures, immobilization with a plaster cast of the leg may be required for ~4–6 weeks, while for arthroscopic surgeries, it is typically around 3–4 weeks to allow for the complete healing of the SPR. Hence, the overall rehabilitation time may be extended for open surgeries.^13^

### Clinical Evaluation

Collect patient data multiple times throughout the treatment process, including before surgery, 1 month after surgery, at 3 months post‐operation, and during the final follow‐up (at least 6 months). The clinical outcomes were evaluated using preoperative and postoperative scores and an MRI of SPR. The score included the Visual Analog Scale (VAS), American Orthopaedic Foot and Ankle Society score (AOFAS), ankle eversion muscle strength, Pain Interference score (PI), and Physical Function (PF) of the Patient‐Reported Outcomes Measurement Information System (PROMIS) score. The two surgical groups were compared within themselves to analyze the clinical outcomes (intragroup comparison), and the clinical outcomes of the four time periods were also compared between the two groups (intergroup comparison).

### Muscle Strength Measurement

**FIGURE 5:**
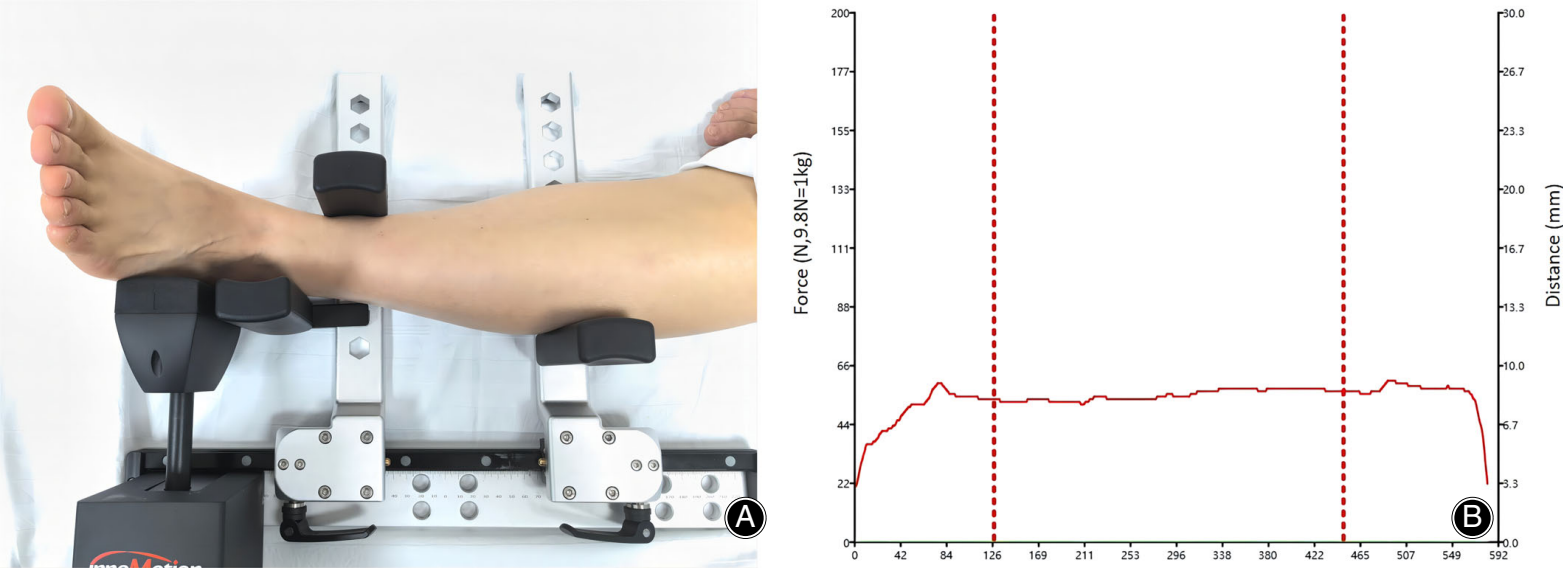
(A) Positioning for muscle strength measurement; (B) Illustration of muscle strength measurement data (the data between the two red dashed lines are considered valid data).

Peroneus muscle strength was measured by the Ligs Joint Ligament Digital Body Checker (InnoMotion, Shanghai, China). The instrument was purchased in 2021, and we only conducted muscle strength measurements on 36 patients, with 16 patients in the arthroscopic group and 20 patients in the open group, and we improved the usage method of the instrument only to record the values of the pressure applied by the muscles to the instrument. The subjects were positioned in a supine position with their tested leg extended and the tibia kept horizontal. The positional component was adjusted to secure the tibia and ankle joint. The device handle was pushed to the lateral side of the foot and locked in place. The subjects actively everted the ankle joint while the pressure exerted by the foot on the handle was recorded for 15 s. Ligs software (InnoMotion, Shanghai, China) was used to extract valid data within the recorded time and calculate the average value (Figure 5).

### Statistics

The data were analyzed using IBM SPSS version 22.0 (IBM, Armonk, NY, USA). The Kolmogorov–Smirnov test was used to confirm a normal distribution. The mean ± standard deviation represents data conforming to a normal distribution. For intragroup mean comparisons, a one‐way analysis of variance (ANOVA) was employed, followed by post hoc LSD tests for multiple comparisons. The nonnormally distributed measurement data are represented by M (Q1, Q3) and were compared using the Kruskal–Wallis test, followed by Dunn's post hoc multiple comparison tests. For intergroup mean and demographics comparisons, a paired t test or Mann–Whitney U test was employed. A significance level of p < 0.05 would indicate statistically significant differences.

## Results

### Comparison of Demographics between Two Groups

Forty‐six patients met the criteria, including 35 males and 11 females, and 26 patients underwent open surgery, while 20 patients underwent arthroscopic surgery. Among the cases, 44 were attributed to sports injuries, while two were caused by falls. The time of illness for the open surgery group was 6 (5, 11) months, with a follow‐up time of 10 (8, 15) months and a surgical time of 47.73 ± 7.50 min. For the arthroscopic surgery group, the time of illness was 8 (6, 9) months, with a follow‐up time of 10 (8, 11.75) months and a surgical time of 85.70 ± 13.23 min. The comparison of surgical time between the two groups showed a statistically significant difference (p < 0.01) (Table 1).

**TABLE 1:** Comparison of demographics between arthroscopic and open surgery group.

Group	Number	Age (year)	BMI (kg/m^2^)	Time of illness (months)	Time of follow‐up (months)	Surgical time (minutes)
Open surgery	26	22 (18, 25)	26.98 (24.45, 28.61)	6 (5, 11)	10 (8, 15)	47.73 ± 7.50
Arthroscopic	20	20.5 (18, 24, 75)	24.98 (22.52, 27.74)	8 (6, 9)	10 (8, 11.75)	85.70 ± 13.23
Statistic value (t/Z)		−0.5	−1.041	−0.545	−0.96	−11.494
p		0.617	0.298	0.586	0.339	<0.01

Abbreviation: BMI, Body mass index.

### Postoperative Complications

**FIGURE 6:**
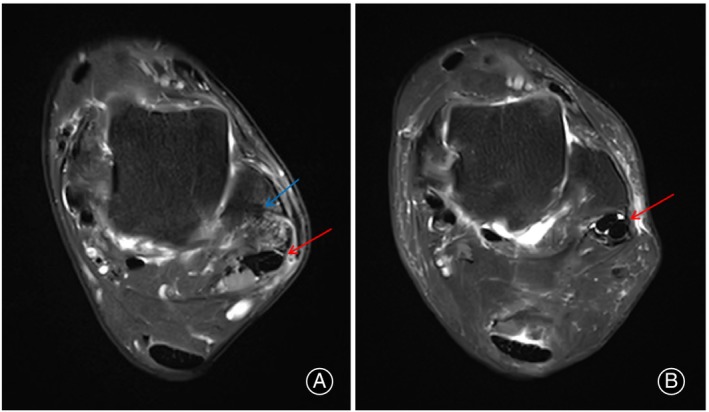
Postoperative MRI. (A) Position of suture anchor (blue arrow) and knot of wire (red arrow); (B) Complete continuity of SPR (red arrow).

During the surgical procedures, groove‐deepening was performed in two cases of open surgery and four cases of arthroscopic surgery. Following the operation, all patients underwent postoperative MRI reexamination, which showed restoration of the SPR (Figure 6). Notably, out of the patients who underwent open surgery, one experienced sural nerve symptoms, which notably improved after the stitch was removed. Furthermore, three patients who underwent open surgery experienced limitations in ankle dorsiflexion and pain; however, with intensified rehabilitation exercises, their symptoms improved by the final follow‐up. In another case involving arthroscopic surgery, a patient developed peroneal tendon subluxation, but saw symptom improvement by the final follow‐up after strengthening the peroneal muscles and using ankle supports.

### Intragroup Comparison of the Two Groups

In the open surgery group, the AOFAS score increased from 76.50 ± 9.18 (preoperative) to 91.73 ± 6.55 (final follow‐up), and the PF score increased from 48.5 (46.75, 50) (preoperative) to 60 (55, 73) (final follow‐up). The VAS score decreased from 3 (2, 3) (preoperation) to 1 (0.75, 2) (6 months), and the PI decreased from 55 (52, 58) (preoperation) to 44 (41.25, 47.25) (final follow‐up). Based on the above data, we can conclude that patients showed significant improvement in functional and pain scores at final follow‐up postoperatively (p < 0.01). However, it is important to note that the open surgery group exhibited significant improvement in functional (AOFAS and PF) and pain scores (VAS and PF) only at final follow‐up postoperatively. Moreover, the data indicate that muscle strength did not recover to the preoperative level (7.17 [5.21, 7.84]) within the first 3 months after surgery (3.63 [2.96, 4.43]). It took final follow‐up postoperatively (6.93 [5.38, 7.47]) for the muscle strength to finally return to the baseline level observed before the operation (Table 2).

**TABLE 2:** Intragroup comparison and intergroup comparison of arthroscopic and open surgery group.

Group	AOFAS		VAS		PI		PF		Muscle strength (kg)	
	A (n = 26)	B (n = 20)	A (n = 26)	B (n = 20)	A (n = 26)	B (n = 20)	A (n = 26)	B (n = 20)	A (n = 20)	B (n = 16)
Preoperative	76.50 ± 9.18^d^	75.5 (71.25, 82)^c,d^	3 (2, 3)^d^	3 (2,3)^c,d^	55 (52, 58)^d^	54.5 (52, 57)^c,d^	48.5 (46.75, 50)^c,d^	47.5 (41, 49.75)^c,d^	7.17 (5.21, 7.84)^b,c^	6.89 ± 1.96^b^
1 month postoperative	77.85 ± 4.57^d^	76.5 (72.25, 81.75)^c,d^	3 (2, 3)^d^	2 (2, 3)^c,d^	53 (52, 55)^d^	51 (49.25, 55)^c,d^	48.5 (48, 50)^c,d^	49 (47.25, 51)^a,c,d^	2.93 (2.19, 3.62)^a,d^	3.44 ± 1.51^a,c,d^
3 month postoperative	80.23 ± 7.16^d,^ *	87 (83, 87)^a,b,^ *	2 (2, 2)^d,^ *	1 (1, 2)^a,b,^ *	53 (48, 56)^d,^ *	44 (43.25, 48)^a,b,^ *	51 (50, 53)^a,d,^ *	60 (53, 73)^a,b,^ *	3.63 (2.96, 4.43)^a,d,^ *	5.12 ± 1.14^b,*^
Final follow‐up	91.73 ± 6.55^a,b,c^	90 (87,100)^a,b^	1 (0.75, 2)^a,b,c^	1 (0.25, 1)^a,b^	44 (41.25, 47.25)^a,b,c^	43.5 (39.25, 47)^a,b^	60 (55, 73)^a,c^	73 (55, 73)^a,b^	6.93 (5.38, 7.47)^c,b,^ *	7.66 ± 1.80^b,*^
Statistic value (F/H)	25.137	53.344	41.246	41.730	42.244	43.701	50.894	50.072	46.958	21.469
p	<0.01	<0.01	<0.01	<0.01	<0.01	<0.01	<0.01	<0.01	<0.01	<0.01

Note: Group A = Open surgery, group B = Arthroscopic surgery; a,b,c,d, respectively represent statistically significant comparisons with preoperative, 1 month postoperative, 3 months postoperative, and final follow‐up.

Abbreviations: AOFAS, American Orthopaedic Foot and Ankle Society score; PI, Pain Interference score; PF, Physical Function (PF); VAS, Visual Analog Scale.

* Represent statistical differences in comparisons between different groups at the same time.

In arthroscopic patients, the AOFAS score increased from 75.5 (71.25, 82) (preoperative) to 90 (87, 100) (final follow‐up), and the PF score increased from 47.5 (41, 49.75) (preoperative) to 73 (55, 73) (final follow‐up). The VAS score decreased from 3 (2, 3) (preoperative) to 1 (0.25, 1) (final follow‐up), and the PI decreased from 54.5 (52, 57) (preoperative) to 43.5 (39.25, 47) (final follow‐up). Based on the above data, we can conclude that patients showed significant improvement in functional and pain scores at final follow‐up, and this improvement was statistically significant (p < 0.01). Additionally, muscle strength reached the preoperative baseline level (6.89 ± 1.96) within 3 months postoperatively (5.12 ± 1.14) (Table 2).

### Intergroup Comparison of the Two Groups

After 3 months postoperatively, the arthroscopic surgery group showed superior functional scores (AOFAS = 87 [83, 87], PF = 60 [53, 73]) compared to the open surgery group (AOFAS = 80.23 ± 7.16, PF = 51 [50, 53]), p < 0.01. The arthroscopic surgery group also had lower pain scores (VAS = 1 [1, 2], PI = 44 [43.25, 48]) compared to the open surgery group (VAS = 2 [2], PI = 53 [48, 56]), p < 0.01. At 3 months postoperatively and final follow‐up, the arthroscopic surgery group exhibited superior muscle strength (5.12 ± 1.14, 7.66 ± 1.80) compared to the open surgery group (3.63 [2.96, 4.43], 6.93 [5.38, 7.47]), p < 0.01.

## Discussion

There is a wide array of treatment approaches to tackle PTD, and the predominant emphasis within existing literature has been on case reports and descriptions of surgical techniques. However, comparative studies examining the outcomes of surgical interventions are notably rare. The primary objective of our study is to furnish empirical evidence to guide the selection of surgical procedures for Ogden types 1–2 PTD. Our research has revealed that, during the initial 3 months following surgery, there were notable differences in muscle strength, pain levels, and functional recovery between patients undergoing open surgery versus those undergoing arthroscopic surgery. However, by the 6‐month postoperative milestone, both surgical approaches appeared to provide similar outcomes, though muscle strength remained somewhat lower in the open surgery group when compared to the arthroscopic group. The study supports the viability of arthroscopic surgery for patients requiring early postoperative exercise and the maintenance of structural integrity of SPR.

### Limitations and Complications of Open Surgical Approaches for PTD

According to existing literature, the leading open surgical approaches can be categorized into four types: 1. SPR repair, 2. Local tissue reinforcement of the SPR, 3. Peroneal tendon‐bone blocking technique, 4. Groove‐deepening technique.^14^, ^15^ Our team has been utilizing these methods to treat PTD since 2017, and long‐term follow‐up has shown positive clinical outcomes. However, we have also identified certain limitations associated with this surgery, including an extended recovery period, restricted ankle joint mobility, and symptoms related to the sural nerve. In our study, we observed that only at the 6‐month postoperative mark did the functional and pain scores of the open surgery group demonstrate significant improvement. Furthermore, it took 6 months following the surgery for the muscle strength to recover to the baseline level observed before the operation (Table 2). During our 26 open surgery cases, we encountered some specific complications such as sural nerve irritation and ankle dorsiflexion pain postsurgery. To address this issue, the author has identified several potential reasons: 1. Aggravation of original SPR and fibular fibrous ridge damage: Open surgery for Ogden1‐2 type PTD may unintentionally worsen the damage to the original SPR and the fibular fibrous ridge. This increased damage could contribute to a longer recovery time for the patients. 2. Proximity of the incision to the sural nerve: The incision's location behind the fibula in the surgical procedure is close to the sural nerve. Furthermore, the overlapping suture method used during the procedure can stimulate symptoms in the sural nerve. 3. The tightness of the sutures in the SPR can also be a potential reason. During surgery, if the suture knot is excessively tightened, it can inadvertently reduce the space within the peroneal tendon sheath. This decrease in space can increase the sliding resistance of the tendon and cause additional pressure on the SPR. Ultimately, this can impact the postoperative function and recovery of patients.^7^, ^16^

### Advantages of Arthroscopic Surgery for PTD

Arthroscopy, introduced by Wertheimer in 1995, has been proven to be an effective method for both diagnosing and treating foot and ankle diseases.^8^, ^9^ This minimally invasive surgical approach offers several advantages, including reduced trauma, less postoperative pain, faster recovery, shorter hospital stays, and a decreased risk of sural nerve injury.^4^ Our study found that patients who underwent arthroscopic surgery achieved functional and pain scores within the normal range 3 months after the procedure accompanied by a significant improvement in muscle strength. Furthermore, there was no significant difference compared to 6 months postsurgery (Table 2). The authors believe that arthroscopic examination does not compromise the integrity of the SPR, which is why early participation in patient rehabilitation exercises is encouraged. In addition to the benefits above, arthroscopy enables the surgeon to observe the sliding motion of the peroneal tendon within its sheath, allowing for real‐time adjustments to the tension of the SPR repair, ensuring optimal outcomes. Moreover, the irrigation function of arthroscopy helps prevent the formation of fibrosis around the tendon, improving postoperative healing and overall function.^17^, ^18^

### Limitations of Arthroscopic Surgery and the Necessity of Groove‐Deepening

**FIGURE 7:**
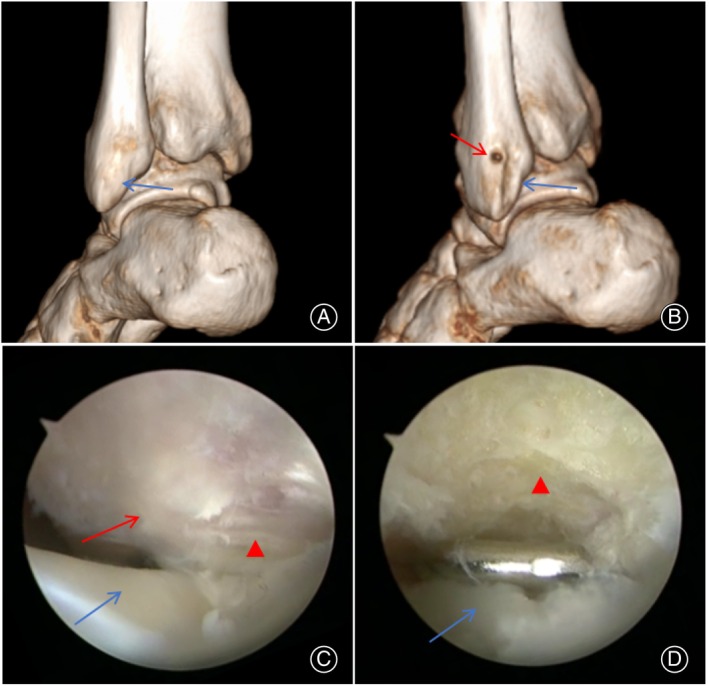
(A) Preoperative CT showed that the fibular groove was flat (blue arrow). (B) Postoperative deepening of the fibular groove (blue arrow) and the position of the anchor (red arrow). (C) Flat fibular groove (red arrowhead), SPR (red arrow), and peroneus longus tendon (blue arrow) under arthroscopy. (D) Deepening groove (red arrowhead) and peroneus brevis tendon removed the low‐lying muscle belly (blue arrow).

In treating PTD, arthroscopic and open surgical procedures follow the same guiding principles. These principles include creating more space for tendon movement, relieving lower muscle compression on the SPR, and reducing healing tension to repair the SPR. However, the methods used in these two surgical approaches differ. Some scholars hold the viewpoint that the stable function of the peroneal tendon relies on the integrity of the SPR and have raised questions about the necessity of performing groove‐deepening surgery.^19^ Park suggested that PTD is primarily caused by trauma, and spontaneous dislocation is rare. Simple repair of the SPR without groove deepening can yield favorable clinical outcomes.^15^ Adachi reported that the morphology and shape of the fibular groove were not significantly different in patients with traumatic PTD, and groove‐deepening surgery may even hinder smooth sliding of the tendon.^20^ Maffulli also stated that the low incidence of PTD indicates that the groove is not a predisposing factor for dislocation.^15^ The author acknowledges the importance of the SPR in stabilizing the peroneal tendon. However, we cannot fully agree with the scholars' viewpoints above, as they did not consider the limitations of arthroscopic surgery. First, in arthroscopic surgery, the placement of the suture anchor is typically anterior to the fibular fibrous ridge (Figures 6 and 7). This suturing method does not provide anatomical repair and carries the risk of peroneal tendon subluxation. Therefore, one patient in the arthroscopic group experienced subluxation in our study. Additionally, remnants of the SPR can form pseudo cysts that cannot be completely excised arthroscopically, leading to a decrease in the overall strength of the SPR.^8^ Last, it is challenging to address PTD caused by SPR avulsion fractures through arthroscopic procedures. Therefore, groove‐deepening procedures can increase the tendon sliding space and promote SPR healing.^21^ During surgery, we closely observed the overall condition of the fibular ridge and groove. One specific method involves placing a probe parallel to the groove and proceeding with groove deepening when there is no significant curvature (Figure 7). There is no denying that arthroscopic surgery has significant limitations in terms of patient selection. Candidates for arthroscopic surgery must meet specific criteria, such as good tendon quality, intact SPR, and relatively normal joint anatomy. On the other hand, open surgery offers more selectivity and is more suitable for patients with severe tendon injury, poor SPR quality, or Ogden III‐IV type injuries.

### Limitations and Prospect

This study has several limitations: it is a retrospective cohort study with a limited number of cases and a short follow‐up duration. Additionally, not all patients underwent muscle strength testing and there was no measurement of muscle strength on the healthy side compared to the affected side for study. Future investigations should include larger sample sizes and employ prospective designs to evaluate the biomechanical changes and clinical outcomes associated with both surgical methods to ensure more accurate comparisons.

## Conclusion

For patients with PTD of Ogden type 1–2, arthroscopic surgery offers greater advantages; however, it is undeniable that open surgery has a wider range of selection. Nevertheless, regardless of the type of PTD, arthroscopy should be the first choice. This approach allows for a comprehensive evaluation of the integrity of the SPR and the presence of other tendinopathies of the peroneal tendon. Relying solely on MRI, B‐ultrasound, and physical examination may not provide a complete picture, and arthroscopy can offer valuable insights to guide the most suitable surgical decision.^14^, ^19^, ^22^

## Author Contributions

Zhenyu Wang and Xu Tao: wrote the main manuscript text. Fangcheng Yang, Guo Zheng, Yuanqiang Li, Yang Liu, Xinyu Xie: conducted data analysis and collection.

## Funding Information

This work was supported by the Chongqing Science and Health Joint Medical Research Project (2023MSXM121) and the Sports Injury Repair and Reconstruction Research Innovation Group (cstc2020jcyj‐cxttX0004).

## Conflict of Interest Statement

The authors declare that they have no conflicts of interest.

## Ethics Statement

This study was performed in line with the principles of the Declaration of Helsinki. Approval was granted by the Ethics Committee of the First Affiliated Hospital of Army Medical University ((B)KY2023151).
